# Strawberry-Based Cosmetic Formulations Protect Human Dermal Fibroblasts against UVA-Induced Damage

**DOI:** 10.3390/nu9060605

**Published:** 2017-06-14

**Authors:** Massimiliano Gasparrini, Tamara Y. Forbes-Hernandez, Sadia Afrin, Patricia Reboredo-Rodriguez, Danila Cianciosi, Bruno Mezzetti, Josè L. Quiles, Stefano Bompadre, Maurizio Battino, Francesca Giampieri

**Affiliations:** 1 Dipartimento di Scienze Cliniche Specialistiche ed Odontostomatologiche (DISCO)-Sez. Biochimica, Facoltà di Medicina, Università Politecnica delle Marche, 60131 Ancona, Italy; m.gasparrini@univpm.it (M.G.); tamara.forbe@gmail.com (T.Y.F.-H.); dolla.bihs@gmail.com (S.A.); preboredo@uvigo.es (P.R.-R.); danila.cianciosi@gmail.com (D.C.); 2 Area de Nutrición y Salud, Universidad Internacional Iberoamericana (UNINI), 24040 Campeche, Mexico; 3 Departamento de Quimica Analıtica y Alimentaria, Grupo de Nutricion y Bromatologıa, Universidade de Vigo, 32004 Ourense, Spain; 4 Dipartimento di Scienze Agrarie, Alimentari e Ambientali, Università Politecnica delle Marche, 60131 Ancona, Italy; b.mezzetti@univpm.it; 5 Department of Physiology, Institute of Nutrition and Food Technology “José Mataix”, Biomedical Research Centre, University of Granada, 18000 Granada, Spain; jlquiles@ugr.es; 6 Dipartimento di Scienze Biomediche e Sanità Pubblica, Facoltà di Medicina, Università Politecnica delle Marche Via Ranieri 65, 60131 Ancona, Italy; s.bompadre@univpm.it; 7 Centre for Nutrition & Health, Universidad Europea del Atlantico (UEA), 39011 Santander, Spain

**Keywords:** skin damage, UVA-radiation, ROS, apoptosis, mitochondria functionality, antioxidant defense, inflammatory status, strawberry polyphenols, cosmetic formulation

## Abstract

Extreme exposure of skin to Ultraviolet A (UVA)-radiation may induce a dysregulated production of reactive oxygen species (ROS) which can interact with cellular biomolecules leading to oxidative stress, inflammation, DNA damage, and alteration of cellular molecular pathways, responsible for skin photoaging, hyperplasia, erythema, and cancer. For these reasons, the use of dietary natural bioactive compounds with remarkable antioxidant activity could be a strategic tool to counteract these UVA-radiation-caused deleterious effects. Thus, the purpose of the present work was to test the efficacy of strawberry (50 μg/mL)-based formulations supplemented with Coenzyme Q_10_ (100 μg/mL) and sun protection factor 10 in human dermal fibroblasts irradiated with UVA-radiation. The apoptosis rate, the amount of intracellular reactive oxygen species (ROS) production, the expression of proteins involved in antioxidant and inflammatory response, and mitochondrial functionality were evaluated. The results showed that the synergic topical use of strawberry and Coenzyme Q_10_ provided a significant (*p* < 0.05) photoprotective effect, reducing cell death and ROS, increasing antioxidant defense, lowering inflammatory markers, and improving mitochondrial functionality. The obtained results suggest the use of strawberry-based formulations as an innovative, natural, and useful tool for the prevention of UVA exposure-induced skin diseases in order to decrease or substitute the amount of synthetic sunscreen agents.

## 1. Introduction

Even if ultraviolet (UV) radiation possesses some health benefits, such as the stimulation of cholecalciferol production, and is often used to cure some skin pathologies, such as vitiligo and psoriasis, it remains the principal cause of different skin disorders [1,2]. The main pathological effects of UV radiation range from erythema and premature aging to cancer; at the same time, it is able to induce cellular modifications such as alterations in elastic fibers and collagen, loss of subcutaneous adipose tissue, and photo-carcinogenic changes [1]. UVA makes up 95% of incident light and is more penetrating than UVB, reaching the subcutaneous tissue, and affecting both dermal and epidermal skin structures [3]. Exposure to UVA radiation can, in fact, lead to several biological phenomena, including inflammation, oxidative stress, damage to DNA, and signaling pathway dysregulation, mainly through the production of ROS [4]. The harmful effects of UV radiation on the skin are generally counteracted through the use of topical sunscreen products [5,6]. Sunscreens protect the skin against cancer and they also prevent the onset of other skin diseases caused by solar radiation, such as wrinkle formation, collagen loss, undesired pigmentation, and aging. Indeed, the topical application of sunscreens represents an effective precautionary measure against these problems mainly in regions with high solar radiation levels [1]. Recently, compounds derived from natural sources have attracted remarkable attention for their implementation in sunscreen products and have encouraged the market trend towards natural cosmetics. This aspect underlines the importance of identifying an extensive selection of natural active molecules in sunscreen formulations, in order to reduce the quantity of synthetic sunscreen agents present in cosmetic formulations [5]. For these reasons, in the last few years, protective properties have been studied for diverse natural polyphenols, including luteolin, silymarin, grape seed proanthocyanidins, green tea polyphenols, genistein, and strawberry anthocyanins [7,8]. Strawberries (*Fragaria X ananassa*, Duch.) are a considerable source of minerals, vitamins, sugars, and polyphenols such as anthocyanins, phenolic acids, and flavonoids [9,10]. Altogether, these bioactive molecules have synergistic activities on the promotion of health and the prevention of different diseases, such as aging, cardiovascular diseases, inflammatory-related pathologies, and cancer [11,12,13]. In particular, we recently evidenced that polyphenols and vitamins present in strawberries exert an effective protection against skin damage induced by oxidative- and UVA-stressors, reducing DNA damage, lipid peroxidation, free radical levels, and improving mitochondrial functionality when added in the culture medium, and increasing cell-viability when combined in a sunscreen formulation [6,8,14]. Here, we used a specific combination of a strawberry extract enriched with coenzyme Q_10_ (CoQ_10_) in a cosmetic formulation to counteract UVA’s damaging effects on human dermal fibroblasts (HDF), assessing apoptosis rate, ROS intracellular production, the level of antioxidant and inflammatory marker proteins, and mitochondria functionality.

## 2. Materials and Methods

### 2.1. Standard and Reagents

UV-filters samples were kindly supplied by BASF (Cesano Maderno, Italy): bis-ethylhexyloxyphenol methoxyphenyl triazine (Tinosorb S Aqua, active), diethylamino hydroxybenzoyl hexyl benzoate (Uvinul A Plus) and Octocrylene (Uvinul N539T). Liquid CoQred (Quinomit^®^) was kindly provided by MSE Pharmazeutika GmbH (Bad Homburg, Germany). All chemicals and solvent were of analytical grade. Cyanidin (Cy)-3-glucoside, Pelargonidin (Pg)-3-glucoside, ferrous sulphate (FeSO_4_), 2,20-azino-bis-(3-ethylbenzothiazoline-6-sulfonic acid) diammonium salt (ABTS), 2,2-diphenyl-1-picrylhydrazyl (DPPH), 6-hydroxy-2,5,7,8-tetramethyl-chroman-2-carboxylic acid (Trolox), oligomycin, 2,4-dinitrophenol (2,4-DNP), antimycin A, rotenone and all other reagents and solvents were purchased from Sigma-Aldrich chemicals, Milan, Italy. CellROX^®^ Orange reagent was purchased from Invitrogen^TM^, Life Technologies, Milan, Italy. Dulbecco’s Modified Eagle Medium (DMEM), were obtained from Carlo Erba Reagents, Milan, Italy, as well as all other products for cell cultivation. Primary and secondary antibodies were purchased from Santa Cruz Biotechnology, Dallas, TX, USA and Bioss Inc., Woburn, MA, USA and all the other products for western blot analysis from Bio-Rad Laboratories, Inc., Hercules, CA, USA.

### 2.2. Strawberry Samples

Strawberry fruits (*Fragaria × ananassa*, Alba variety) were collected in the experimental fields of the Agricultural Faculty of Università Politecnica Marche; within 2 h after harvest, whole fruits were stored at -20°C before analyses and subjected to methanolic extraction as previously described [14]. The total phenolic content (TPC) of strawberry extract was determined by the Folin–Ciocalteu method [15], total flavonoid content (TFC) by the aluminium chloride spectrophotometric method [16], while vitamin C (vit C) and folate content were analyzed by a HPLC system [17,18]. Anthocyanins (ACYs) solid-phase extraction and HPLC-MS/MS analysis were performed as previously described [19]. Finally, total antioxidant capacity (TAC) was determined using Trolox Equivalent Antioxidant Capacity (TEAC) [20], Ferric Reducing Antioxidant Power (FRAP) assays [21] and the 2,2-DiPhenyl-1-PicrylHydrazyl free radical method (DPPH) [22]. For filter preparation, strawberry extract was concentrated under vacuum at 40 °C to eliminate total methanol and suspended in the final formulation. Results were reported as mean value of three replicates ± standard deviation (SD).

### 2.3. Filter and Formulation Preparation

A UV filter combination often used in sun protection factor 10 (SPF10) sunscreen products was chosen for this study and prepared as follows: 2% bis-ethylhexyloxyphenol methoxyphenyl triazine, 2% diethylamino hydroxybenzoyl hexyl benzoate and 2% Octocrylene. According to our preliminary viability data [6], this formulation (SPF10 group) was enriched with (i) Alba strawberry extract at 50 µg/mL (Strw group); (ii) reduced CoQ_10_ (CoQ_10red_ group) at 100 µg/mL and (iii) a combination of strawberry extract + CoQ_10red_ (Strw+CoQ_10red_ group).

### 2.4. Cell Culture

HDF were Human Dermal Fibroblasts isolated from adult skin obtained from the American Type Culture Collection (Manassas, VA, USA). HDF were plated into a T-75 flasks and cultured as previously described [6]. Cells were maintained in a HeraCell CO_2_ incubator at 37 °C with 5% CO_2_ and the medium was changed every 2–3 days. All the tests were conducted on cells between the 4^th^ and the 6th passage. 

### 2.5. UVA Treatment

**Figure 1:**
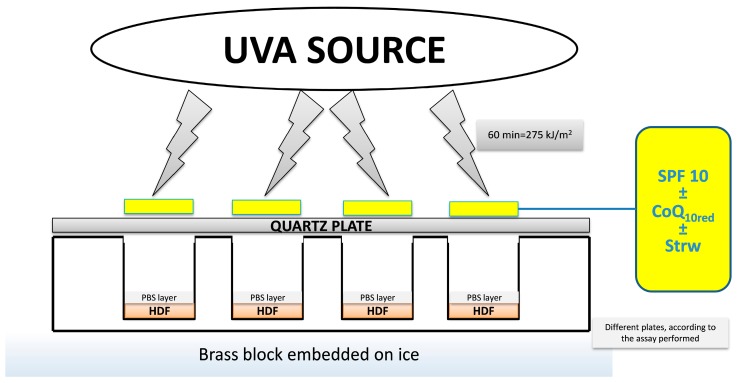
Schematic representation of the experimental setup used to apply the different formulations to the cell cultures. UVA, Ultraviolet A; PBS, phosphate buffered saline; HDF, Human Dermal Fibroblast; SPF 10, sun protection factor 10; CoQ_10red_, reduced coenzyme Q_10_ and Strw, strawberry.

As previously described [6], a Saalmann Palma Plant Box Universal (Biosonic, Bologna, Italy) equipped with a 320 W ozone-free lamp, UV type 3, was used as UVA irradiating source. The source delivered 15 mW/cm^2^ between 300 and 400 nm at a distance of 20 cm from the cell cultures; it was always pre-run for 10 min to allow the output to stabilize. The incident dose of UVA received by the samples was 275 kJ/m^2^ (60 min of exposure), i.e., a dose approximately equivalent to about 90 min of sunshine at the French Riviera (Nice, France) in summer at noon [23]. Cells grown on different plates, depending on the analysis to be performed, were washed twice with phosphate buffered saline (PBS) and covered with a thin layer of PBS prior to exposure. Each formulation (2 mg/cm^2^) was spread onto quartz-bottom petri dishes (Hubei Yunsheng Quarts Products Co., Ltd., Xiaogan Hanchuan, China) of exactly the same dimensions as the cell culture plates and the dishes were placed on top of the wells prior to irradiation. The cells were placed on a brass block embedded in ice in order to reduce the temperature and hence the evaporation during exposure, which could eventually dry out the medium. The cells were then either not exposed (UV−), or exposed to the UVA source (UV+) as previously described for 60 min (Figure 1), because longer exposure times showed a significant loss of vitality.

### 2.6. Apoptosis Detection

Apoptosis was measured using the Tali™ apoptosis assay kit and propidium iodide (Invitrogen™, Life Technologies, Milan, Italy) as previously reported [24]. Concisely, on the first day of the assay 1.5 × 10^5^ cells were seeded in a 6-well plate and left to adhere for 16–18 h. The day after seeding, the cells were subjected to UV radiation, with different SPF10-enriched filter combinations, as previously described. At the end of the treatment cells were centrifuged (1500 rpm for 5 min), and resuspended in 100 μL of annexin binding buffer (ABB). After that, 5 μL of Annexin V Alexa Fluor^®^ 488 was added, mixed well, and the solution was incubated in the dark at room temperature for 20 min. Then cells were centrifuged at 1500 rpm, resuspended in 100 μL of ABB, and 1 μL of propidium iodide was added, mixed well, and incubated in the dark at room temperature for 5 min. Samples were analyzed using the Tali^®^ Image-Based cytometer and the percentage of apoptotic nuclei, dead cells, and live cells was determined on the basis of the corresponding fluorescence histogram compared with an untreated control. The results were expressed as fold increase compared with the control.

### 2.7. Intracellular ROS Concentration

Intracellular ROS generation was determined by CellROX^®^ Orange Reagent (Invitrogen™, Life Technologies, Milan, Italy) as previously described [25]. On the first day of the assay, 1.5 × 10^5^ cells were seeded in a 6-well plate, and left to adhere for 16–18 h. The day after seeding, the cells were subjected to UV radiation, with different SPF10-enriched filter combinations, as previously described. At the end of the treatment, the medium was removed and collected, then CellROX^®^ Orange Reagent, a fluorogenic probe for measuring oxidative stress in live cells, was added (1:500 dilution) to 1 mL of DMEM. This cell-permeant dye is non-fluorescent while in a reduced state and exhibits bright fluorescence upon oxidation by ROS. Samples were incubated at 37 °C for 30 min, centrifuged at 320× *g*, and then resuspended in PBS. Cells were analyzed with the Tali^®^ Image-Based cytometer and unexposed cells were used to determine baseline levels of intracellular ROS and to set the fluorescence threshold for the Tali^®^ instrument. The results were expressed as fold increase compared with the control.

### 2.8. Evaluation of Mitochondria Functionality

Oxygen consumption rate (OCR) was measured in real-time using a XF-24 Extracellular Flux Analyzer (Seahorse Bioscience, Billerica, MA, USA), as previously reported [26]. Briefly, cells were seeded for 16 h in the XF-24 plate before the UVA treatment with different SPF10-enriched filter combinations, as previously described. At the end of the treatment, the medium was replaced with 450 µL/well of XF-24 running media (supplemented with 25 mM glucose, 2 mM glutamine, 1 mM sodium Pyruvate, without serum) and pre-incubated at 37 °C for 20 min in the XF Prep Station incubator (Seahorse Bioscience, Billerica, MA, USA) in the absence of CO_2_. The plate was then transferred to the XF-24 Extracellular Flux Analyzer and after an OCR baseline measurement, a profiling of mitochondrial function was performed by sequential injection of four compounds that affect bioenergetics, as follows: 55 µL of oligomycin (2.5 µg/mL) at injection in port A, 61 µL of 2,4-dinitrophenol (2,4-DNP) (1 mM) at injection in port B, and 68 µL of antimycin A/rotenone (10 µM/1 µM) at injection in port C. The best concentration of each inhibitor and uncoupler was obtained on the basis of a proper titolation curve. The final results were expressed as pmol of O_2_ consumed per 10^5^ cells per min (pmol O_2_/10^5^ cells/min). Moreover, the Maximal Respiratory Capacity value of each treatment was calculated with the following equation [27]:

Maximal Respiratory Capacity = (2,4-dinitrophenol OCR value − antimycin A/rotenone OCR value)
(1)

Also in this case, the final results were expressed as pmol O_2_/10^5^ cells/min.

### 2.9. Immunoblotting Assay

After UVA exposure with different filter-enriched combinations, cells were collected, washed with PBS, lysed in 100 μL lysis buffer (120 mmol/L NaCl, 40 mmol/L Tris [pH 8], 0.1% NP40), and centrifuged at 13,000× *g* for 15 min. An immunoblotting assay was performed as previously described [26]. Proteins from cell supernatants were separated on a 10–15% acrylamide SDS/PAGE (Bio-Rad, Hercules, CA, USA). Proteins were transferred onto a nitrocellulose 0.2 μm membrane (Bio-Rad, Hercules, CA, USA) using the trans-blot SD semidry electrophoretic transfer cell (Bio-Rad, Hercules, CA, USA) and then membranes were blocked with TBS-T containing 5% non-fat milk for 1 h at room temperature. Membranes were incubated at 4 °C overnight with the primary antibody solution, diluted at 1:500 (*v*/*v*), specific for the detection of: nuclear factor kappa-light-chain-enhancer of activated B cells (NF-kB), phosphorylated nuclear factor of kappa light polypeptide gene enhancer in B-cell inhibitor, alpha (pIκBα), interleukin (IL)-6, IL-1β, tumor necrosis factor alpha (TNF-α), nuclear factor E2-related factor 2 (Nrf2), catalase, superoxide dismutase (SOD), and heme oxygenase 1 (HO-1). The GAPDH protein was used for the measurement of the amount of protein analyzed. Then, membranes were probed for 1 h at room temperature with their specific alkaline phosphatase-conjugated secondary antibodies (1:80,000 dilution *v*/*v*). Immunolabeled proteins were detected by using a chemiluminescence method (CDiGit Blot Scanner, LI-COR, Bad Homburg, Germany). Quantification of gene expression was carried out using the software provided by the manufacturer of the Blot Scanner (Image Studio 3.1). The protein concentration was determined by the Bradford method [28]. Data were expressed as fold increase compared to control.

### 2.10. Statistical Analysis

Each analysis was carried out in triplicate and the results were given as mean ± standard deviation (SD). Data between different groups were analyzed statistically using the one-way ANOVA and Turkey’s post hoc test. Statistical analyses were performed using STATISTICA software (Statsoft Inc., Tulsa, OK, USA). *p* < 0.05 was considered as significant.

## 3. Results and Discussion

### 3.1. Analysis of Strawberry Fruits

As already reported in our previous studies [6,25,26], the Alba cultivar contained a good amount of polyphenols (TPC), flavonoids (TFC) and vitamin C (vit C), with values of 2.52 mg Gallic Acid Equivalent/g fresh weight (FW), 0.66 mg Catechin Equivalent/g FW, and 0.58 mg vit C/g FW, respectively (Table 1). HPLC-DAD/ESI-MS analysis allowed us to detect five anthocyanin pigments, with Pelargonidin (Pg) 3-glucoside (39.74 mg/100g FW) and Pg 3-malonylglucoside (6.69 mg/100g FW) representing the most relevant components (Table 1). Alba extract showed total antioxidant capacity (TAC) values of 22.64, 7.71, and 22.85 µmol Trolox Equivalent/g FW for Trolox Equivalent Antioxidant Capacity (TEAC), 2,2-DiPhenyl-1-PicrylHydrazyl (DPPH) and for Ferric Reducing Antioxidant Power (FRAP), respectively, confirming the results obtained with other strawberry varieties (Table 1) [8,14,29]. Finally, the Alba cultivar possessed 0.99 µg of folinic acid calcium salt hydrate/g FW and 0.06 µg of 5-methyltetrahydrofolic acid/g FW (Table 1): these folate compounds are responsible, synergistically with other strawberry bioactives, for several health effects, as previously highlighted by different authors [30,31].

### 3.2. Apoptosis Rate Regulation by Different Formulation

**Figure 2:**
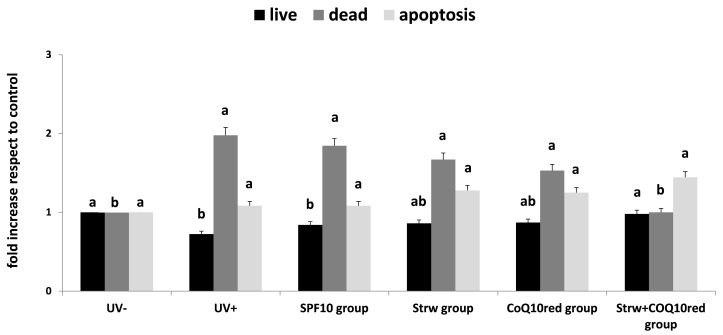
Live, dead, and apoptosis levels in human dermal fibroblast (HDF) cells irradiated with ultraviolet A (UVA, 275 kJ/m^2^) radiation (UV+) or not irradiated (UV−), after 1 h of exposure. HDF were screened with different formulations as explained in the text. Data are expressed mean values ± standard deviation (SD). Columns with different superscript letters are significantly different (*p* < 0.05).

UV irradiation can promote different harmful effects including DNA damage, sunburn, and apoptosis [32]. Apoptosis is widely considered as a major mechanism of regulated cell death which is defined by distinct morphological and biochemical features and exerts a key role in embryogenesis, aging and tissue homeostasis. The ability of different bioactive compounds to modulate the life or death of a cell is recognized as a tool of therapeutic potential [5,32]. Consequently, many existing treatments have been developed which may act through the regulation of apoptosis [24,33]. In our work, we first confirmed the results previously obtained (Figure 2) [6]: the combined action of strawberry and CoQ_10red_ significantly reduced the level of dead cells compared to UV exposed cells (*p* < 0.05), restoring the level of live cells to values similar to the control group (UV−) (*p* < 0.05). Taking into account the apoptosis rate, no significant differences were detected between the tested groups, even if the apoptotic cell level increased with Strw, CoQ_10red_ and Strw + CoQ_10red_ treatments. These results, with the concomitant reduction of dead cells, could be interpreted as a possible protective mechanism exerted by Strw and CoQ_10_, which delay cellular death in UV-exposed cells. As far as we know, this is the first study which explores the apoptotic effects of strawberries on UV-exposed HDF, however further studies will be necessary to confirm these results.

### 3.3. Protection of Different Formulations Against ROS Accumulation

**Figure 3:**
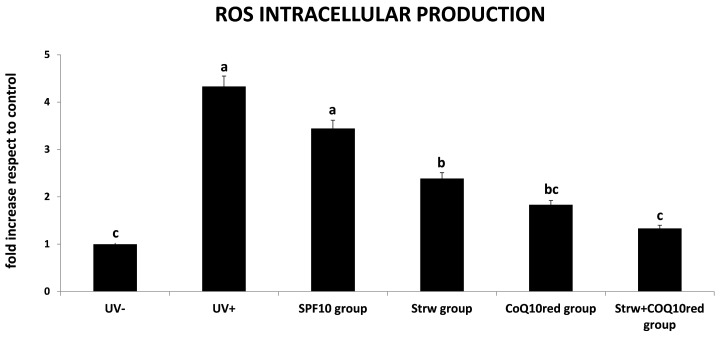
Reactive oxygen species (ROS) level in human dermal fibroblast (HDF) cells irradiated with ultraviolet A (UVA, 275 kJ/m^2^) radiation (UV+) or not irradiated (UV−), after 1 h of exposure. HDF were screened with different formulations as explained in the text. Data are expressed mean values ± standard deviation (SD). Columns with different superscript letters are significantly different (*p* < 0.05).

Inflammation, immunosuppression and DNA damage are the main consequences of UV radiation exposure. These effects are directly and indirectly caused by high levels of ROS which destabilize biomolecules and induce chain reactions leading to membrane degradation, mitochondrial damage, telomere shortening and deterioration, and oxidation of enzymatic and structural proteins [4]. Oxidative stress induced by ROS is also the primary cause of photoaging and also promotes cell death and cancer [2]. For this reason, the measurement of ROS production could be a valuable tool to estimate UVA-induced oxidative damage. In our work, the protective effect of the different cosmetic formulations was demonstrated (Figure 3). In HDF cells subjected to UV radiation (UV+), a significant increase in the amount of ROS was detected, compared to untreated cells (UV−) (*p* < 0.05). This effect was efficiently counteracted through the different formulations applied: a significant difference (*p* < 0.05) compared to UV-exposed cells was detected in the Strw group and the CoQ_10red_ group. Interestingly, the maximal protective effect was found in the Strw + CoQ_10red_ group (*p* < 0.05), suggesting an important synergic action. These data obtained with this berry were in line with previous results found using a topical pretreatment of green tea polyphenolic extract in a human model [34], and with other berry and natural bioactive compounds applied in different cellular [35,36,37,38,39] and animal models [40,41] exposed to UV radiation.

### 3.4. Effect of Different Fomulations on Mitochondrial Respiration Rate

**Figure 4:**
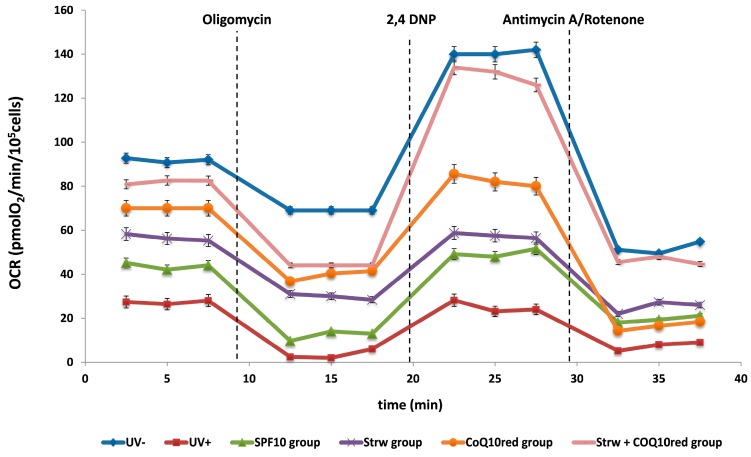
Oxygen consumption rate (OCR) value of human dermal fibroblast (HDF) cells irradiated with ultraviolet A (UVA, 275 kJ/m^2^) radiation (UV+) or not irradiated (UV−), after 1 h of exposure. HDF were screened with different formulations as explained in the text. Data are expressed as mean values ± standard deviation (SD).

The most important site of ROS production is the mitochondrial electron transport chain (ETC) [33]. In this process, in fact, a small percentage of electrons directly reacts with oxygen, causing the formation of ROS as secondary ETC products [33]. As previously reported, UV irradiation causes mutations to mitochondrial DNA, altering its function by reducing the consumption of O_2_ and the production of ATP, which affects cell migration and division [4,42]. UV exposure promotes mitochondrial dysfunction and toxicity mainly through the activation of caspases, the release of cytochrome C, and the depolarization of the membrane. Additionally, mitochondrial dysfunction augments the oxidative stress levels at mitochondrial complexes [4]. In this context, we investigated the implication of mitochondrial dysfunction in HDF cells after UVA radiation in association with the different protective formulations, through OCR measurement. Basal respiration is mainly driven by the concomitant re-entry pathways through the proton leak and ATP synthases. After that, cells were exposed consecutively to four modulators of oxidative phosphorylation, such as oligomycin, 2,4-DNP, and antimycin/rotenone. Oligomycin stops the ATP synthase so that residual respiration is due solely to the proton leak. The addition of the protonophore 2,4 DNP causes an artificial proton conductance into the membrane. This maximal respiration is now regulated by the activity of the ETC and/or the delivery of substrate. The maximal respiratory capacity is defined as the increased respiratory capacity above basal respiration. At the end, ETC inhibitors were added: antimycin A/rotenone that block complex III and I, respectively. In this way, any residual respiration is nonmitochondrial and needs to be subtracted from the other rates. In Figure 4, the trend of the different treatments was shown as a function of the different inhibitors applied. Basal OCR was noticeably damaged in cells subjected to UV radiation (UV+). On the contrary, the different formulations applied to screen the cells improved mitochondrial functionality, increasing the OCR value and restoring values to levels similar to those of unexposed cells (UV−) in Strw + CoQ_10red_ group (Figure 3).

**Figure 5:**
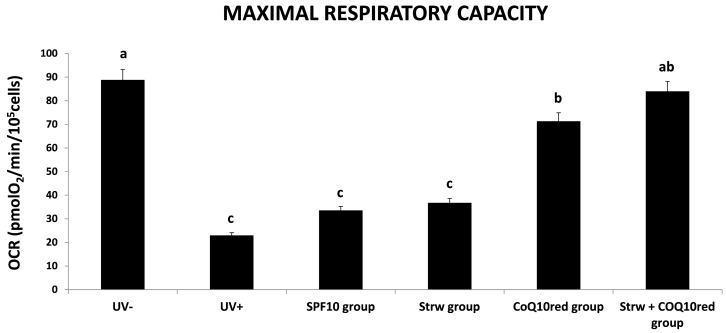
Maximal respiratory capacity value of human dermal fibroblast (HDF) cells irradiated with ultraviolet A (UVA, 275 kJ/m^2^) radiation (UV+) or not irradiated (UV−), after 1 h of exposure HDF were screened with different formulations as explained in the text. Data are expressed as mean values ± standard deviation (SD). Columns with different superscript letters are significantly different (*p* < 0.05).

Taking into account the maximal respiratory capacity (Figure 5), UV-irradiated cells showed the lowest values, while the different protective formulations applied raised this rate, in particular in the Strw + CoQ_10red_ group, which presented a value similar to unexposed cells (*p* < 0.05).

As far as we know, there are no published studies that underline the effect of different sunscreen formulations against UV irradiation in HDF cells, especially regarding OCR results. For this reason, further investigations are needed to clarify and investigate the possible protection mechanisms exerted on mitochondrial functionality.

### 3.5. Different Formulations Positively Regulated Antioxidant and Inflammatory-Related Pathways

**Figure 6:**
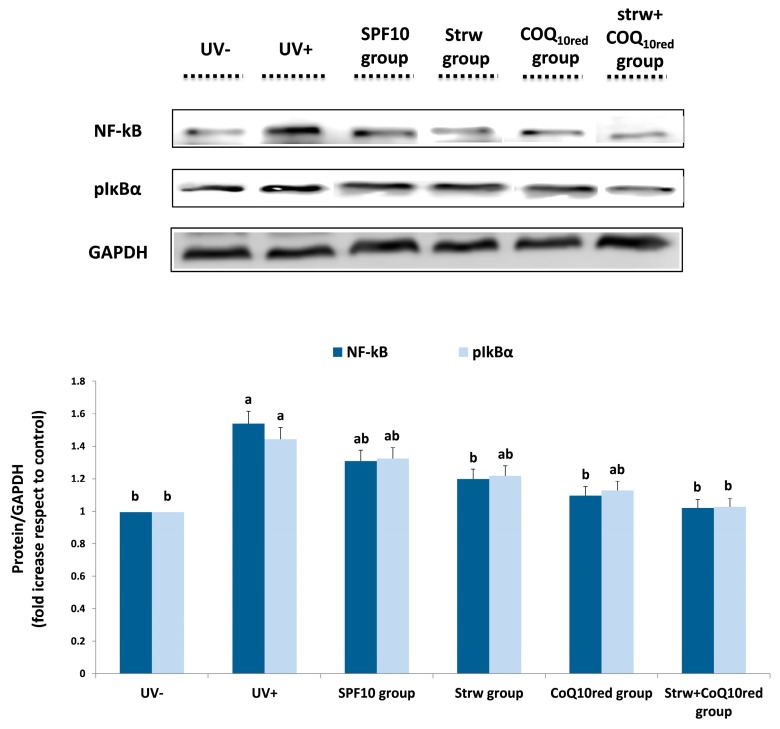
Levels of inflammatory markers (NF-kB, pIkBα) in human dermal fibroblast (HDF) cells irradiated with ultraviolet A (UVA, 275 kJ/m^2^) radiation (UV+) or not irradiated (UV−), after 1 h of exposure. HDF were screened with different formulations as explained in the text. Data are expressed as mean values ± standard deviation (SD). Columns belonging to the same set of data with different superscript letters are significantly different (*p* < 0.05).

**Figure 7:**
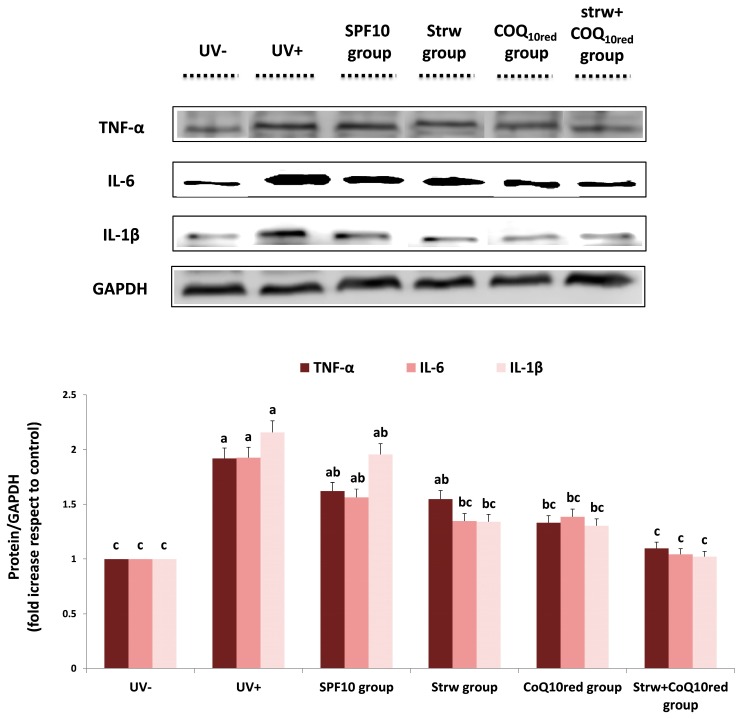
Levels of inflammatory markers (TNF-α, IL-1β, IL-6) in human dermal fibroblast (HDF) cells irradiated with ultraviolet A (UVA, 275 kJ/m^2^) radiation (UV+) or not irradiated (UV−), after 1 h of exposure. HDF were screened with different formulations as explained in the text. Data are expressed as mean values ± standard deviation (SD). Columns belonging to the same set of data with different superscript letters are significantly different (*p* < 0.05).

Estimation of the antioxidant and anti-inflammatory effects of the different enriched formulations on different molecular pathways involved in cellular response was performed by protein expression analysis. UV radiation activates pro-inflammatory genes which represent significant mediators of photocarcinogenesis and the photoaging processes, such as IL-6 and TNF-α [4]. The transcriptional factor that controls the inflammatory response is NF-κB which plays a crucial role in the carcinogenesis and inflammation induced by UV exposure [43]. In the cytoplasm, NF-kB is in an inactive form due to complexation with IkBα. In response to inflammatory stimuli, after IkBα phosphorylation (to form pIkBα), NF-kB is activated and migrates to the nucleus, up-regulating inflammation-related genes such as pro-inflammatory cytokines [26]. In our work, the different formulations applied reduced the levels of the tested inflammatory markers (NF-kB, pIkBα, TNF-α, IL-6, and IL-1β), whose expression was considerably increased after UV-exposure (*p* < 0.05) (Figure 6 and Figure 7). In the case of NF-kB and pIkBα, a significant reduction in protein expression was registered with CoQ_10red_ and Strw + CoQ_10red_ enriched formulations, respectively (*p* < 0.05) (Figure 6). Similar results were obtained for IL-6 and IL-1β: also in this case the efficient reduction provided by the Strw-enriched formulation (*p* < 0.05) was enhanced by the combination with CoQ_10red_. Finally, in the case of TNF-α, a value similar to the control group (UV−) was obtained with thebCoQ_10red_-enriched formulation (*p* < 0.05) (Figure 7). Interestingly, in all the proteins investigated, the combination of Strw and CoQ_10red_ provided the best results in terms of inflammatory marker reduction (Figure 6 and Figure 7). These data are in line with the results obtained in previous studies, in which the expression of NF-kB and pro-inflammatory cytokines induced by UV radiation was improved through treatment with different bioactive compounds in dermal keratinocytes and fibroblast cells [37,44,45,46]

**Figure 8:**
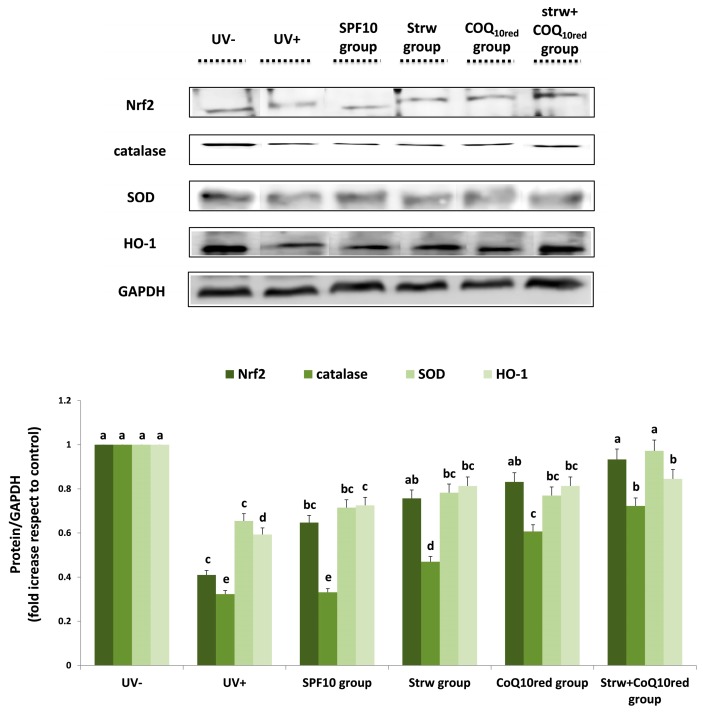
Levels of proteins related to antioxidant response (Nrf2, catalase, SOD, HO-1) in human dermal fibroblast (HDF) cells irradiated with ultraviolet A (UVA, 275 kJ/m^2^) radiation (UV+) or not irradiated (UV−), after 1 h of exposure. HDF were screened with different formulations as explained in the text. Data are expressed as mean values ± standard deviation (SD). Columns belonging to the same set of data with different superscript letters are significantly different (*p* < 0.05).

Nrf2 is also targeted by UV radiation. This protein is the main regulator of cellular antioxidant defense, since it regulates the expression of many “antioxidant responsive elements” (ARE), which positively regulate a wide array of antioxidant and detoxification enzymes involved in cellular defense against oxidative stress, such as catalase, SOD, and HO-1 [26]. Nrf2 also regulates the bioavailability of mitochondrial respiration, highlighting the role of bioenergetics in antioxidant protection and in cell structure stabilization [4]. In our work, the different enriched formulations applied increased Nrf2 levels, restoring values similar to control (UV−) levels, especially in the Strw + CoQ_10red_ group (*p* < 0.05) (Figure 8). An analogous trend was detected with the other genes investigated (Figure 8). In the case of SOD, an efficient improvement with respect to UV-exposed cells was obtained with the Strw group (*p* < 0.05), restoring values similar to unexposed cells in the Strw + CoQ_10red_ group (*p* < 0.05) (Figure 8). Similar results were obtained for catalase and HO-1 (Figure 8), even if the protein expression level of the unexposed cells was never reached. These results confirm previous data, which highlights the protective mechanism of plant phenolic compounds against UV-induced damage through Nrf2 activation [47], and the improvement of antioxidant reserves [48,49]. All the collected evidence underlines the idea that strawberry-enriched sun-protective formulations were able to counteract the UV-mediated inflammatory response by acting on the different pathways. The different formulations efficiently reduced the inflammatory conditions created by UV radiation through the augmentation of antioxidant defense expression, the modulation of the NF-kB pathway, and the reduction of inflammatory cytokine production.

## 4. Conclusions

There is a global tendency towards the use of natural cosmetics and to create high UV-protection formulations with little amounts of chemical substances. The combination of synthetic agents and natural compounds may offer an effective tool for preventing the damaging effects of UV exposure. In our work, the use of strawberry/reduced CoQ_10_-based formulations has been proposed as an innovative instrument for the prevention of UVA exposure-induced inflammation. The obtained results suggest the possible applications of this natural screen against dermal damage caused by UV radiation, as highlighted by the reduction of ROS and pro-inflammatory markers, and the improvement of mitochondrial functionality and antioxidant enzyme expressions. Further research is strongly encouraged to deeply investigate the molecular mechanisms involved, and to find the main classes of bioactive compounds responsible for this anti-inflammatory effect.

## Figures and Tables

**Table 1:** Phenolic content and antioxidant capacity of Alba strawberry extract. Data are presented as mean value ± SD. TPC, total polyphenols content; TFC, total flavonoids content; vit C, vitamin C; ACYs, anthocyanins; Cy-3-glucoside, cyanidin-3-glucoside; Pg 3-glucoside, pelargonidin 3-glucoside; Pg 3-rutinoside, pelargonidin 3-rutinoside; Pg 3-malonylglucoside, pelargonidin 3-malonylglucoside; Pg 3-acetylglucoside, pelargonidin 3-acetylglucoside; TAC, total antioxidant capacity; TEAC, Trolox Equivalent Antioxidant Capacity; DPPH, 2,2-DiPhenyl-1-PicrylHydrazyl and FRAP, Ferric Reducing Antioxidant Power.

Parameter	Mean Value
TPC (mg GAEq/g FW)	2.52 ± 0.01
TFC (mg CEq/g FW)	0.66 ± 0.01
vit C (mg vit C/g FW)	0.58 ± 0.02
ACYs (mg/100g FW)	
Cy-3-glucoside	3.11 ± 0.03
Pg 3-glucoside	39.74 ± 0.13
Pg 3-rutinoside	3.87 ± 0.16
Pg 3-malonylglucoside	6.69 ± 0.04
Pg 3-acetylglucoside	0.39 ± 0.01
TAC (µmol Teq/g FW)	
TEAC	22.64 ± 0.49
DPPH	7.71 ± 0.32
FRAP	22.85 ± 0.39
Folate (µg folate/g FW)	
folinic acid calcium salt hydrate	0.99 ± 0.09
5-methyltetrahydrofolic acid	0.06 ± 0.01
